# Racial/Ethnic Trends in Virtual Mental Health Care Utilization Among Undergraduate Students: A Descriptive Study

**DOI:** 10.1007/s40615-024-02028-7

**Published:** 2024-05-23

**Authors:** D. M. Loren, T. Reuter, N. Bickham, R. T. Booth

**Affiliations:** TimelyCare, 1315 S Adams St., Fort Worth, TX 76104 USA

**Keywords:** Mental health treatment disparities, Young adults, College students, Virtual mental health

## Abstract

**Background:**

Undergraduate students are particularly in need of mental health support, but demand has far surpassed resources. This gap between mental health diagnoses and support is particularly large among Asian, Hispanic/Latinx, and Black students. Supplementing on-campus care with a virtual-only behavioral health partner may shift these trends.

**Objective:**

This study is aimed at comparing the number of undergraduate students from different racial/ethnic groups (White, Asian, Islander, Hispanic/Latinx, Black, Native, and Multiracial) engaging in virtual mental health visits as part of a partnership with a company providing virtual-only care, with the total enrolled undergraduate students at the same 113 institutions.

**Methods:**

We used de-identified visit data and self-reported race/ethnicity to define the “patient” population of undergraduates accessing care. We compared that to the full “student” population of undergraduates among the same schools, available as part of the Integrated Postsecondary Education Data System (IPEDS).

**Results:**

Patient population race/ethnicity (*N* = 14,870) differed significantly from student population race/ethnicity (*N* = 619,459). A significant effect ($\chi$^2^_6_ = 2258, *P* < .001) indicated that patient demographics differed from student demographics. We found proportionally *more* Asian, Black, and Multiracial patients than students. At the same time, we found proportionally *fewer* White and Hispanic/Latinx patients than students.

**Conclusions:**

We conclude that, in contrast to prior literature in traditional mental health care, some racial/ethnic minority undergraduates (Asian, Black, and Multiracial) may actually access care at a *higher* rate under a fully virtual model. On the other hand, White and Hispanic/Latinx students may access care *less* frequently.

## Introduction

Mental health concerns among college-aged adults have been on the rise for several decades [1], with one recent study finding a 50% increase in mental health diagnoses among this age group from 2013 to 2021 [2]. Furthermore, access to mental health treatment in this group has historically lagged behind need, with 26% of young adults perceiving an unmet need in mental health services in the past 12 months [3]. This problem is present across all college-age youth, including those at public and private universities, and may be particularly significant among community college students, where rates of diagnoses are higher and access is even lower [4]. Improving access to care in the college student population is paramount for addressing the rising rates of mental health issues. Unfortunately, in the past 10 years, the need for support has increasingly eclipsed on-campus resources [5], leaving a gap that has recently been filled by college and university partnerships with supplemental virtual mental health care providers [6]. 

### Racial/Ethnic Differences

This emergence of virtual mental health care offerings on college campuses offers several exciting opportunities for exploration. In this article, we aim to examine one such opportunity: the possibility of shifting demographic trends in access to mental health care among college students relating to the arrival of a fully virtual care option.

Research shows that historically, access to mental health care among college students has differed greatly by demographics, including race/ethnicity: on the whole, non-White college students have accessed mental health care at lower rates than White college students [2]. Unfortunately, care utilization rates are not reflective of the rates of mental health concerns among these groups. Some studies estimate that rates of mental health concerns are similar among White and non-White students [7], while others find that non-White students likely bear a *higher *burden of mental health concerns due to historic discrimination and systemic racism [8], implying they should be accessing care at higher rates in order to receive an equally adequate level of support. Specifically, extensive work has documented the negative impact of racial microaggressions on the mental health of Asian-American, Hispanic/Latinx, Black, Native, and Multiracial individuals [9, 10], including college students [11]. Other unsavory aspects of American society also increase the likelihood of negative mental health outcomes among non-White college students, with just a few examples being (1) constantly changing migration policy and fear of deportation among undocumented Hispanic/Latinx students [12, 13] and (2) the salience of the “historical loss” of homeland, people, and culture, due to colonization, among Native students [14]. The recent COVID pandemic has been hypothesized to only exacerbate these negative societal impacts on minority mental health, with noteworthy increases in blatant discrimination against Asian-American students likely leading to increased mental health concerns [15, 16]. Therefore, any disparities in access to mental health care only serve to further drive disparities in outcomes. Below, we present a literature review of historical rates of mental health treatment access in college or college-aged youth among racial/ethnic groups.

Notably, below, we benchmark rates of mental health care access among college students with the most recent relevant publication from the Healthy Minds study, which examines college student mental health and treatment-seeking using a large, national survey dataset. For reference throughout the below sections, in the most recent Healthy Minds data, *55.8% of White students* with one or more mental health conditions sought care over the 2020–2021 data collection period, the highest access rate among the racial/ethnic groups in this year and nearly all of the seven prior years. For context, rates of screening positive for one or more mental health problems ranged from 50.4 to 67.3% across groups, with 60.8% of all White students screening positive for one or more mental health problems in the 2020–2021 data collection period.

#### Asian-American

Findings often indicate that access to mental health care among young adults is lowest for those who identify as Asian-American. This is in keeping with prior work examining access to care among the broader category of Asian-American adults, wherein this group is the least likely to access care overall [17, 18]. Among college students, the Healthy Minds study utilizes a demographic category labeled “Asian Pacific Islander Desi American” (APIDA) and found that in 2020–2021, only *33.8% of APIDA students* with one or more mental health problems had sought mental health treatment of any kind, the lowest of any demographic group [1]. For context, 58.4% of all APIDA students screened positive for one or more mental health problems. Another study proposed even lower numbers, finding that only 20% of Asian young adults with apparent mental health conditions accessed care, and hypothesized cultural stigma as the driver of this low ratio [7]. Several other studies have supported the hypothesis that stigma and acculturation underlie this underutilization of mental health care among Asian-American college students [19, 20]. Interestingly, language of care is a documented barrier in the literature on disparities in mental health care access among Asian-American adults [21, 22] but is not cited as a barrier among the young adult/college population. Notably, for our study, one study examined mental health among Pacific Islander students specifically, suggesting similar barriers among these students, including stigma, language barriers, and cost of care [23]. 

#### Hispanic/Latinx

Hispanic/Latinx students may be the second-least likely racial/ethnic group to access care for mental health conditions. Among the literature documenting access among Hispanic/Latinx adults more broadly, researchers find a significant and increasing gap between Hispanic/Latinx and White adults’ access to mental health care [17, 24]. In college students specifically, the Healthy Minds survey data indicated that *35.9% of Latinx students* (labeled “Latino/a/@” in their study) with one or more mental health conditions accessed care over the 2020–2021 data collection period, out of the 62.1% of all Hispanic/Latinx students who screened positive for one or more condition. Several studies have examined potential causes for the underutilization of mental health care among Hispanic/Latinx students: one such study found mental health care usage was negatively correlated with spirituality [25], while another found stigma to be the most prominent barrier [26]. Language barriers have also been more extensively studied and suggested to drive increasing disparities in care access among Hispanic/Latinx adults [17, 21, 27], but there is less work to suggest that these barriers hold among Hispanic/Latinx young adults/college students.

#### Black

Many studies find that Black young adults access care at lower rates than White young adults [28]. This effect has been well-documented in the literature among Black adults more broadly, with studies finding an increase in the access disparity over time [17]. One framework designed to capture the barriers to mental health care among Black adults in the USA points toward stigma, cost, and lack of transportation/childcare [29]. Among college students specifically, in the most recent Healthy Minds study data, *37.7% of Black college students* with one or more mental health conditions accessed care over the 2020–2021 data collection (out of 50.4% of all Black students who screened positive for one or more condition), placing them as the third-lowest access group [2]. Researchers have hypothesized that a multitude of factors may act as barriers to black students accessing mental health care, including stigma, structural racism, microaggressions, and familial and spiritual influences [30, 31]. 

#### Native

Although there is somewhat less literature documenting and exploring disparities in access to care among Native American individuals, epidemiologic data suggest that Native individuals may access mental health care at rates below White individuals but in line with national averages [32]. The literature among Native American adults highlights that barriers to care access are broad and rooted in a lack of cultural diversity and inclusion in the mental health care system [33] and may include preferring to use informal over formal supports, structural obstacles, stigma/shame, and uncertainty and mistrust about what services would entail [34]. Among college students more specifically, the Healthy Minds survey data indicates that *48.3% of Native college students* (in a group they refer to as “American Indian/Alaska Native”) with one or more mental health conditions accessed care between 2020 and 2021 [2]. For context, 63.3% of all Native students screened positive for having one or more mental health conditions. Unfortunately, we are not aware of any scholarly work specifically examining barriers to access among Native college students.

#### Multiracial

Finally, it is important to note that a large and growing percentage of young people in the US identify with more than one racial/ethnic group, making categorization into one of the above groups less and less helpful as a form of distinction. The literature suggests that multiracial young adults merit specific attention [35], with studies finding that multiracial college students were less likely to have sought services or been prescribed medication for mental health than their White peers [36]. Indeed, the Healthy Minds data finds that *50.7% of Multiracial college students* with one or more mental health conditions accessed care from 2020 to 2021, placing them immediately below White students as the group with the greatest access. For context, it is important to note that Multiracial students had the highest rates of *screening positive for* one or more mental health conditions, at 63.3% overall. There is limited research examining barriers to mental health care among Multiracial college students specifically; however, one study suggests that they are less likely to know someone who sought psychological services or who was diagnosed with a psychological disorder compared to their White peers, which may suggest cultural norms and stigma play a role in this community as well [36]. 

### Virtual Care

Due to the recency of shifts toward virtual mental health care among college-aged youth, nearly all of the data supporting the above findings were collected in a time when on-campus in-person care was the prevailing model of support. Therefore, the widespread adoption of virtual settings for the provision of college mental health care during and after the COVID-19 pandemic may have altered utilization patterns. In fact, while examining the first years of the pandemic, researchers found that the usage of telehealth mental health services more than made up for the large decrease in usage of in-person mental health care utilization [37]. Despite this, little is known about to what extent the advent of virtual mental health care may impact the racial/ethnic disparities in access detailed above. Several recent studies and policy reviews have suggested that aspects of virtual care (e.g., enhanced privacy leading to reduced stigma, reduced structural barriers, and greater choice of culture-matched providers) may mean it is one option to attenuate disparities in access [38, 39]; however, it is less frequently empirically studied.

Importantly, one study examining college students did find that, with a tailored online screening and enrollment strategy, students of color were *as likely* to enroll in virtual mental health treatment as non-Hispanic White students, suggesting that this may be an effective avenue to bridging gaps to reach students of color on college campuses [40]. Another study, which did not focus on college students, examined differences in access to (1) in-person, (2) phone, and (3) virtual mental health visits among racial/ethnic groups and found both Black/African American *and* White individuals were more likely than the other studied racial/ethnic groups to utilize phone visits [41]. This suggests that offering remote offerings is more desirable to *certain* demographic groups, although it may differ depending on the specific group of interest. Finally, on the other hand, there is also some work to suggest that, at least in terms of *physical health care*, being of a minority group (specifically Black/African American) actually predicted *less* engagement with a virtual health format [42], suggesting that the likelihood of virtual care reducing access disparities by race/ethnicity may be overestimated.

In light of this scant literature and mixed findings, we designed the present study to empirically clarify the question about whether virtual care shifts demographic access to mental health among college students. We did this by exploring racial/ethnic groups’ usage of mental health visits via TimelyCare, a fully virtual health care provider for college students, as compared to overall demographics on college campuses.

## Methods

### Participants

We aimed to compare demographic data for two groups of participants (patients versus students), matched at the institutional level. *The patient group (n = 14,870)* consisted of all TimelyCare patients who met the following inclusion criteria: (1) an undergraduate student, enrolled at an institution that had demographic data publicly-available via the *Integrated Postsecondary Education Data System* (IPEDS); (2) completed one or more visits via an on-demand virtual emotional support service, a virtual scheduled therapy service, or a virtual psychiatry service between August 1, 2022, and July 31, 2023; (3) self-reported their demographic data during registration. *The student group (n = 619,459)* consisted of all undergraduate students enrolled at one of the 113 IPEDS clients represented by the patient group. In other words, the patient group determined the set of institutions we included in comparative analyses. We necessarily excluded all remaining IPEDS institutions because those schools had no patient data to compare against. Student demographic data were also self-reported and were publicly available via the IPEDS Fall 2022 enrollment dataset.

According to measures from the IPEDS 2022 Fall enrollment dataset, the student group represented a diverse set of USA undergraduate institutions. The sector of the 113 institutions varied, including 25 public, 4-year or above schools, 68 private not-for-profit, 4-year or above schools, 2 private for-profit, 4-year or above schools, and 18 public, 2-year schools. Institution size also varied. Assessing the total students enrolled for credit, analyses included 11 schools with fewer than 1000 students, 51 schools with 1000–4999 students, 22 schools with 5000–9999 students, 14 schools with 10,000–19,999 students, and 15 schools with 20,000 or more students. Institutions also varied in the percent of their full-time first-time undergraduates awarded Pell grants, ranging from 0 to 95% (*M* = 42%, SD = 22%). In sum, the student group represents a broad range of educational contexts, from small liberal arts colleges to large public schools.

### Ethical Considerations

This retrospective study conformed to all guidelines for ethical treatment of participants. All TimelyCare participants sign a Terms and Conditions sheet upon entering into services; this document includes all essential information about TimelyCare’s telemedicine services and privacy policies. We designed this study as an observational, retrospective analysis of data originally collected for quality assurance purposes, and BRANY IRB determined it classified as exempt and waived the requirement for patient consent (Study ID 24-12-097-1665, February 2024). We de-identified and aggregated student data fully prior to delivery to the TimelyCare data science team and secured all patient data on Health Information Portability and Accountability Act (HIPAA)-compliant databases. IPEDS data is publicly available and aggregated; no identifiable data is present.

### Statistical Analysis

We performed these analyses using R Statistical Software (Version 4.3.0) [43]. Reproducible R code is available upon request.

### Power

We completed an a priori power test using the R pwr package [44] to assess whether our sample size would yield adequate statistical power. Results indicate that, using a chi-square test with 6 degrees of freedom and an alpha level of 0.05, a sample size of 6968 participants would be necessary to detect a very small effect size (*w* = 0.05) with 90% power. We therefore concluded that our sample size was appropriate for analyses.

### Data Exclusion

Demographic categories for patients’ and students’ data were broadly aligned. Patients self-reported demographic data during registration, selecting one of nine mutually exclusive categories: American Indian or Native Alaskan, Asian, Black or African American, Middle Eastern/North African (MENA) or Arab Origin, Hispanic or Latino, Native Hawaiian or Other Pacific Islander Native, White, Biracial or Multiracial, or Unknown. IPEDS data is also self-reported and uses eight mutually exclusive categories: American Indian or Alaskan Native, Asian, Black or African American, Hispanic or Latino, Native Hawaiian or Other Pacific Islander, White, Two or More Races, or Unknown. We therefore necessarily excluded 258 patients in the Middle Eastern/North African (MENA) or Arab Origin category because there was no comparable IPEDS demographic category. We also excluded cases where patients and students declined to report their ethnicity (2391 patients and 23,293 students). We consider the limitations of analyzing self-reported demographic data in the "Discussion" section. Despite these exclusions, our remaining sample size remained well above the sample size indicated by our *a priori* power test.

### Analytic Plan

In order to compare patient and student demographics, we aggregated count data for patients and students in each of the eight demographic categories (Table 1). We used a chi-square goodness-of-fit test to compare the demographic characteristics of patients and students. Chi-square tests are non-parametric, meaning they are appropriate when comparison groups have unequal variances and/or data are not normally distributed—cases in which *t*-tests and ANOVAs cannot deliver reliable results [45]. As summarized by Franke and colleagues [46], the chi-square goodness-of-fit test is meant to compare the observed characteristics of a sample against the known characteristics of the population from which that sample is drawn. In the present study, we compared demographics for patients (the sample) versus demographics for students (the population). This omnibus test determines whether or not patient demographics are consistent with student demographics.

Importantly, although the omnibus test indicates whether there is a significant discrepancy between patient and student demographics overall, this test does not indicate which demographic group(s) may be driving the effect. We, therefore, explored which demographic categories might contribute to the omnibus test result by comparing observed and expected counts per cell, following established statistical guidelines for exploratory analyses [47–49]. Results are summarized in Table 2. By comparing demographic distributions for patients and students, these analyses reveal complex patterns of utilization for undergraduate students.

### Hypotheses

Following the above analytic plan, there are three possible hypotheses. First, it is possible that patient and student demographics do not differ significantly, such that the observed and expected number of patients in each demographic category are statistically equivalent. There is limited support for this hypothesis, although the recent adoption of virtual platforms for mental health care means that this work may still be forthcoming. One promising indicator comes from a study indicating that with tailored recruitment/enrollment practices in place, students of color were *as likely* to enroll in virtual mental health treatment as non-Hispanic White students [40]. 

The second hypothesis is that patient and student demographics do differ, such that the observed number of patients in a given demographic category is significantly *less* than the expected number of patients. The second hypothesis therefore predicts that certain groups of patients, perhaps non-White patients, would *under-utilize* virtual mental health care services. The research summarized above lends broad support for this hypothesis for traditional in-person care, finding that non-White students do tend to under-utilize traditional mental health care services due to historic stigma, structural barriers, and use of less formal (peer-based) mental health supports. However, to our knowledge, only one study has found a connection between identifying as Black/African American and being less likely to access *virtual* health care, and it was not examining mental health care specifically [42]. 

The third hypothesis is that patient and student demographics do differ, such that the observed number of patients in a given demographic category is significantly *more* than the expected number of patients. The third hypothesis therefore predicts that certain groups of students, such as non-White students, might *over-utilize* virtual mental health care services. Support for this hypothesis is speculative and based mostly on work proposing virtual health care breaks down barriers to access (stigma, structural racism, etc.) [38, 39], although it has been suggested in one prior study that there was greater utilization of phone visits as opposed to in-person and video visits among both Black/African American individuals and White individuals [41]. 

## Results

**Fig. 1:**
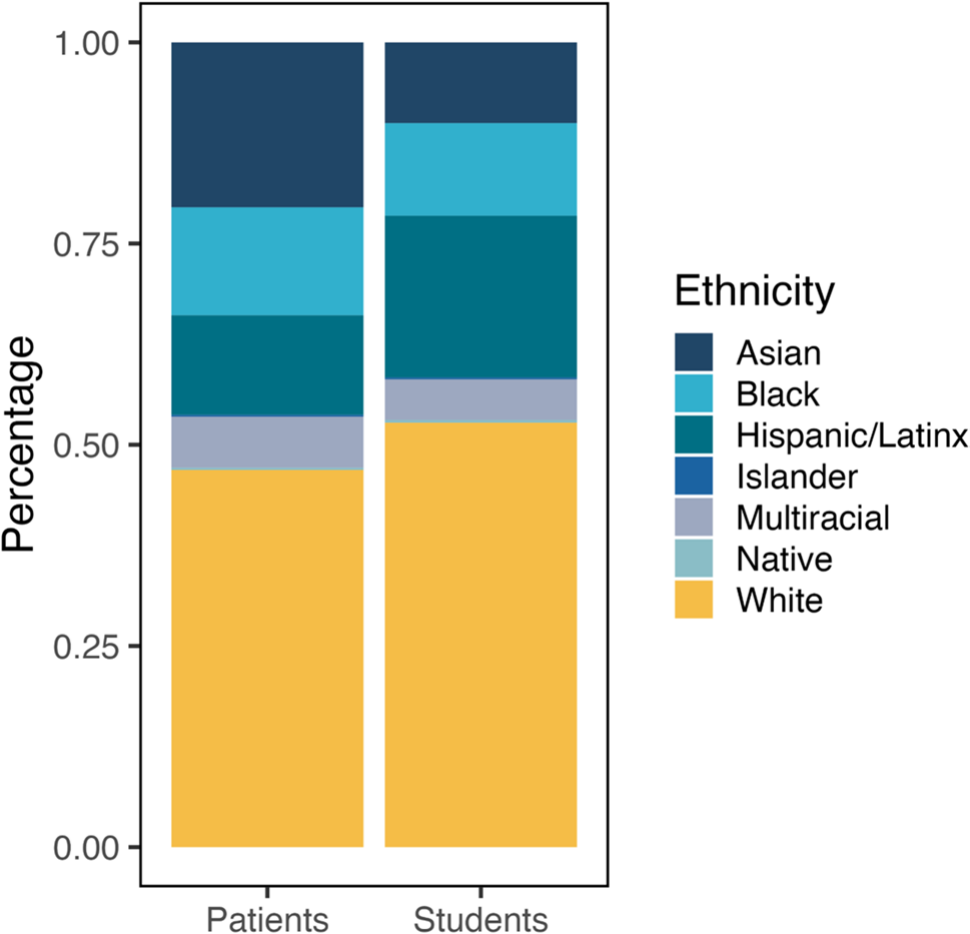
Percent of patients (*N* = 14,870) and students (*N* = 619,459) within each demographic category

A chi-square goodness-of-fit test confirmed that patient demographics differed significantly from student demographics ($\chi$^2^_6_ = 2258, *P* < 0.001). In other words, the observed count of patients per demographic category differed significantly from the expected count of patients per demographic category. This finding implies that undergraduates access virtual mental health care at rates that do not match the overall demographics of the 113 institutions. Visual inspection of the data (Fig. 1) and exploratory statistical analysis (Table 2) revealed a complex pattern of results: We found *more* Asian, Black, and Multiracial patients than expected, given student enrollment. At the same time, we found *fewer* Hispanic/Latinx and White patients than expected. Residuals for Islander and Native students were not significant, such that the observed and expected counts for those categories were statistically equivalent. Thus, rather than aligning with a singular body of prior findings, the present results paint a complicated picture of how undergraduate students engage with virtual mental health care services.

**Table 1:** Count and percent of patients and students within each demographic category

	Patients (*N* = 14,870), *n* (%)	Students (*N* = 619,459), *n* (%)
Asian	3047 (20.49)	62,147 (10.03)
Black	1995 (13.42)	71,279 (11.51)
Hispanic/Latinx	1841 (12.38)	124,509 (20.10)
Islander	34 (0.23)	1402 (0.23)
Multiracial	943 (6.34)	31,259 (5.05)
Native	42 (0.28)	2143 (0.35)
White	6968 (46.86)	326,720 (52.74)

**Table 2:** Exploratory analyses

	Observed count	Expected count	Pearson residuals	Standardized residuals
Asian	3047	1492	40	42
Black	1995	1711	7	7
Hispanic/Latinx	1841	2989	−21	−23
Islander	34	34	0	0
Multiracial	943	750	7	7
Native	42	51	−1	−1
White	6968	7843	−10	−14

The observed count indicates the observed count of patients. The expected count indicates the expected count of patients under the null hypothesis. Pearson residuals are calculated as observed minus expected counts, divided by the square root of expected counts. Standardized residuals are calculated as observed minus expected counts, divided by the square root of the residual cell variance

## Discussion

### Principal Results

This analysis demonstrated that overall, undergraduate students from 113 higher-education institutions access virtual mental health care at rates that do not match the overall demographics of the institutions. In particular, results revealed a *greater proportion of Asian, Black, and Multiracial* undergraduate students utilized this fully virtual mental health care model than would be expected based on the demographics of these institutions. In contrast, we found a *lesser proportion of Hispanic/Latinx and White* undergraduate patients than expected.

Given that prior work demonstrates that access to traditional mental health services has historically been lowest among Asian college students, with Black students not far behind, the first set of findings is striking indeed. They support largely theoretical hypotheses that the barriers to traditional mental health care among this population (e.g., cultural stigma, structural barriers such as childcare and transportation) could be addressed by a virtual model of care like the one studied here.

On the other hand, the finding that a lesser proportion of Hispanic/Latinx and White undergraduate students accessed care is also interesting, especially as Healthy Minds survey data suggest White students are the most likely group to access mental health care, whereas Hispanic/Latinx students are second only to Asian students in being least likely to access care. Examining these two groups together, there may be a common factor of being generally well-represented in the general population (for White students) or in certain geographic locations (for Hispanic/Latinx students). It is therefore possible that some of the hypothesized barriers that would have been removed by virtual care are less present for these groups, who may be able to readily access informal support, or formal mental health care in-person, from someone of their cultural background.

Nonetheless, given the data that Hispanic/Latinx college students have historically under-utilized mental health care, and the hope that this virtual model would alleviate this disparity, the finding is that it did not merit attention. It may be that there are *unique* barriers to care for this group, which are not fully addressed by a virtual-only mental health care solution. One such potential barrier is that of a lack of Spanish-language care through the offering: indeed, our literature review did suggest limited English proficiency as a barrier to care for Hispanic/Latinx adults, although there were no studies to suggest this would be the case for Hispanic/Latinx college students. However, a complication of this interpretation is that language barriers have also been cited among Asian adults and, per our results, do not seem to hold true for Asian college students in this model of care.

The finding that TimelyCare saw fewer Hispanic/Latinx patients than would be expected based on student population did prompt us to consider how the racial/ethnic breakdown of students engaging with this virtual care model might compare to the breakdown of students engaging with on-campus mental health care. We therefore compared our data with the Center for Collegiate Mental Health’s annual report, which includes data gathered from campus wellness centers at over 200 institutions. While the subset of schools did not match the population examined for this study, this data indicates that 59.17% of the students seen in campus mental health centers in the 2022 school year identified as White, while the remaining groups in descending order were 12.03% Asian American, 11.43% Hispanic/Latino/a, 9.92% African American/Black, 5.08% Multiracial, 0.55% American Indian/Alaska Native, and 0.21% Native Hawaiian/Pacific Islander, with 1.61% of students not identifying [50]. Comparing this data qualitatively to Table 1 indicates that the patient breakdown in the present study is even more diverse than that found in aggregate data regarding on-campus mental health centers, including a higher percentage of Hispanic/Latinx students. Therefore, although barriers may not have been entirely removed for this population, this comparison supports the idea that *some* barriers for this population that may still exist in on-campus care are addressed by this fully virtual model.

### Limitations

While important, the present study is not without limitations. We compared aggregate data from TimelyCare visits (patients) with aggregate data from IPEDS (students), matching groups at the institutional level (113 schools). Although we made every effort to ensure valid between-group comparisons, there are notable areas of imprecision. Foremost, all data were self-reported so measures may be inexact within and across groups. We drew students’ demographic data from IPEDS Fall 2022 enrollment, whereas we drew patients’ demographic data from visits completed during the 2022–2023 academic year. While we do not expect a given institution’s demographic makeup to change substantively during a single academic year, it is not possible to know with certainty. Reliability across data sources is similarly uncertain because we cannot determine whether each patient self-reported the same race/ethnicity during virtual care registration as they did during academic enrollment.

A second, related limitation concerns data exclusions. Analyses excluded participants whose degree status was unknown, participants whose race/ethnicity was unknown, and patients who self-reported their ethnicity as MENA as there was no matching category in our comparison dataset. These exclusions were not detrimental to statistical power nor were they likely to have introduced a selection bias effect. However, excluding MENA patients merits a deeper discussion, because disaggregating race/ethnicity categories can reveal important results. For example, one study found Arab Americans had a greater number of COVID-19 cases than previously thought [51]. While it is not possible for us to speculate on what we might find had we been able to separate out individuals identifying as MENA for this paper, equitable research requires high-quality demographic data, and we applaud the United States Office of Management and Budget (OMB) for updating their standards [52], such that future IPEDS data collection will include a MENA category. In the meantime, considering the limitations of self-reported and excluded data, we recommend caution in interpreting these results. The present descriptive study is an important step towards understanding how diverse college students utilize virtual mental health services, but future studies would benefit from directly measuring and comparing (1) student demographics, (2) mental health care access, and (3) *model* of mental health care access (e.g., on-campus, insurance-based, out-of-pocket, virtual, and in-person) within and across institutions. In ongoing research, we aim to compare data from patients and students at an individual level, exploring complex interrelations of mental health, race/ethnicity, and student success.

In addition, due to the aggregate and descriptive nature of the data, we are unable to draw specific causal conclusions about the results. For example, within the IPEDS data, we are unable to identify which of the students included in this study may actually have mental health concerns, limiting our ability to draw conclusions about whether these findings are based on outsized *need* for mental health care or outsized *utilization* of mental health care. However, if our data were driven predominantly by outsized prevalence in *need*, Healthy Minds study data, which also reports the percent of individuals in a given group *who meet criteria for a diagnosis* based on a screening, suggests we would expect that the disproportionate usage would fall among Multiracial, Native, and Hispanic/Latinx students, respectively. This is not the pattern of results we saw, suggesting that this interpretation is not sufficient for explaining the present results. To further clarify this issue, the field would benefit from additional studies that compare student diagnoses with access to mental health care directly in order to more specifically measure *unmet* mental health care needs.

Finally, while we have suggested that the primary differentiator of TimelyCare mental health care from what may be available to students elsewhere (e.g., through insurance, self-pay, or on-campus) is that it is fully virtual, it is also different in other ways, which may drive the present findings. This model is more likely to be covered in terms of cost than some–but not all–of the other options, which may play a role. Additionally, there may be differences in terms of perceived or actual increased diversity of providers available with TimelyCare: counselors at TimelyCare are often staffed/licensed in multiple states, which may lead to a more diverse provider network than some students have access to locally. This may help to reduce stigma and increase utilization for non-White groups. This interpretation is in keeping with significant literature indicating that individuals engaging in mental health care [53], and college students specifically [54], prefer a racial/ethnic match with their therapist. We hope to conduct future studies to directly analyze the relation between college students’ choice to access mental health care in a given setting and the demographic makeup of the available providers in that setting.

## Conclusions

In conclusion, we found significant differences between the racial/ethnic breakdown of more than half a million nationally-represented college students and the racial/ethnic breakdown of those students who chose to access a mental health visit from a fully virtual partner to on-campus services. Specifically, we identified a disproportionately high number of *Asian, Black, and Multiracial students* accessing virtual visits, and a disproportionately low number of *Hispanic/Latinx and White students* accessing virtual visits. These findings are striking given prior literature demonstrating that all groups of non-White college students were less likely to access traditional mental health care and largely support hypotheses that virtual-only mental health care may reduce important barriers to care for non-White students, such as stigma, structural barriers, and a lack of diverse providers. At the same time, findings suggest these barriers may remain among Hispanic/Latinx students, or perhaps that Hispanic/Latinx and White students are accessing care in other ways. Taken together, this study presents promising results for fully virtual mental health care as a way to reduce some of the known racial/ethnic disparities in access among college students, and proposes that offering exclusively virtual mental health care on campus may be an avenue to further diversify who accesses it.

## References

[1] Lipson SK, Lattie EG, Eisenberg D. Increased rates of mental health service utilization by U.S. college students: 10-year population-level trends (2007–2017). Psychiatr Serv Wash DC. 2019;70(1):60–3. 10.1176/appi.ps.201800332. 10.1176/appi.ps.201800332 PMC6408297 30394183

[2] Lipson SK, Zhou S, Abelson S, et al. Trends in college student mental health and help-seeking by race/ethnicity: findings from the national healthy minds study, 2013–2021. J Affect Disord. 2022;306:138–47. 10.1016/j.jad.2022.03.038. 35307411 10.1016/j.jad.2022.03.038 PMC8995361

[3] Cadigan JM, Lee CM, Larimer ME. Young adult mental health: a prospective examination of service utilization, perceived unmet service needs, attitudes, and barriers to service use. Prev Sci. 2019;20(3):366–76. 10.1007/s11121-018-0875-8. 29411197 10.1007/s11121-018-0875-8 PMC6081266

[4] Lipson SK, Phillips MV, Winquist N, Eisenberg D, Lattie EG. Mental health conditions among community college students: a national study of prevalence and use of treatment services. Psychiatr Serv Wash DC. 2021;72(10):1126–33. 10.1176/appi.ps.202000437. 10.1176/appi.ps.202000437 PMC8417151 33657842

[5] Xiao H, Carney DM, Youn SJ, et al. Are we in crisis? National mental health and treatment trends in college counseling centers. Psychol Serv. 2017;14(4):407–15. 10.1037/ser0000130. 29120199 10.1037/ser0000130

[6] Matherly C. Colleges can’t keep up with students’ mental-health needs. Teletherapy companies are filling the gaps. Chron High Educ. Published online January 12, 2024. https://www.chronicle.com/article/colleges-cant-keep-up-with-students-mental-health-needs-teletherapy-companies-are-filling-the-gaps. Accessed 12 January 2024.

[7] Lipson SK, Kern A, Eisenberg D, Breland-Noble AM. Mental health disparities among college students of color. J Adolesc Health. 2018;63(3):348–56. 10.1016/j.jadohealth.2018.04.014. 30237000 10.1016/j.jadohealth.2018.04.014

[8] Liu CH, Stevens C, Wong SHM, Yasui M, Chen JA. The prevalence and predictors of mental health diagnoses and suicide among U.S. college students: implications for addressing disparities in service use. Depress Anxiety. 2019;36(1):8–17. 10.1002/da.22830. 30188598 10.1002/da.22830 PMC6628691

[9] Nadal KL, Griffin KE, Wong Y, Hamit S, Rasmus M. The impact of racial microaggressions on mental health: counseling implications for clients of color. J Couns Dev. 2014;92(1):57–66. 10.1002/j.1556-6676.2014.00130.x.

[10] Choi S, Clark PG, Gutierrez V, Runion C. Racial microaggressions and Latinxs’ well-being: a systematic review. J Ethn Cult Divers Soc Work. 2022;31(1):16–27. 10.1080/15313204.2020.1827336.

[11] Nadal KL, Wong Y, Griffin KE, Davidoff K, Sriken J. The adverse impact of racial microaggressions on college students’ self-esteem. J Coll Stud Dev. 2014;55(5):461–74. 10.1353/csd.2014.0051.

[12] Alif A, Nelson BS, Stefancic A, Ahmed R, Okazaki S. Documentation status and psychological distress among New York City community college students. Cultur Divers Ethnic Minor Psychol. 2020;26(1):11–21. 10.1037/cdp0000290. 31045395 10.1037/cdp0000290

[13] Flores Morales J, Garcia Y. Beyond undocumented: differences in the mental health of Latinx undocumented college students. Lat Stud. 2021;19(3):374–99. 10.1057/s41276-021-00325-4.

[14] Fetter AK, Thompson MN. The impact of historical loss on native American college students’ mental health: the protective role of ethnic identity. J Couns Psychol. 2023;70(5):486–97. 10.1037/cou0000686. 37199954 10.1037/cou0000686

[15] Teng CC, Hon S, Wang A, Tsai W. Impact of COVID-19 discrimination fear on psychological distress among east Asian college students: the moderating role of emotion regulation. Am J Orthopsychiatry. 2023;93(1):86–96. 10.1037/ort0000659. 36355700 10.1037/ort0000659

[16] Wu C, Qian Y, Wilkes R. Anti-Asian discrimination and the Asian-White mental health gap during COVID-19. In: Race and Ethnicity in Pandemic Times. Routledge; 2021.

[17] Cook BL, Trinh NH, Li Z, Hou SSY, Progovac AM. Trends in racial-ethnic disparities in access to mental health care, 2004–2012. Psychiatr Serv. 2017;68(1):9–16. 10.1176/appi.ps.201500453. 27476805 10.1176/appi.ps.201500453 PMC5895177

[18] Abe-Kim J, Takeuchi DT, Hong S, et al. Use of mental health–related services among immigrant and US-born Asian americans: results from the national latino and Asian American study. Am J Public Health. 2007;97(1):91–8. 10.2105/AJPH.2006.098541. 17138905 10.2105/AJPH.2006.098541 PMC1716256

[19] Han M, Pong H. Mental health help-seeking behaviors among Asian American community college students: the effect of stigma, cultural barriers, and acculturation. J Coll Stud Dev. 2015;56(1):1–14. 10.1353/csd.2015.0001.

[20] Kam B, Mendoza H, Masuda A. Mental health help-seeking experience and attitudes in Latina/o American, Asian American, Black American, and White American college students. Int J Adv Couns. 2019;41(4):492–508. 10.1007/s10447-018-9365-8.

[21] Sentell T, Shumway M, Snowden L. Access to mental health treatment by English language proficiency and race/ethnicity. J Gen Intern Med. 2007;22(2):289–93. 10.1007/s11606-007-0345-7. 17957413 10.1007/s11606-007-0345-7 PMC2150610

[22] Kim G, Aguado Loi CX, Chiriboga DA, Jang Y, Parmelee P, Allen RS. Limited English proficiency as a barrier to mental health service use: a study of Latino and Asian immigrants with psychiatric disorders. J Psychiatr Res. 2011;45(1):104–10. 10.1016/j.jpsychires.2010.04.031. 20537658 10.1016/j.jpsychires.2010.04.031

[23] Kwan PP, Soniega-Sherwood J, Esmundo S, et al. Access and utilization of mental health services among Pacific Islanders. Asian Am J Psychol. 2020;11(2):69–78. 10.1037/aap0000172.

[24] Cabassa LJ, Zayas LH, Hansen MC. Latino adults’ access to mental health care. Adm Policy Ment Health. 2006;33(3):316–30. 10.1007/s10488-006-0040-8. 16598658 10.1007/s10488-006-0040-8 PMC2551758

[25] Turner EA, Llamas JD. The role of therapy fears, ethnic identity, and spirituality on access to mental health treatment among Latino college students. Psychol Serv. 2017;14(4):524–30. 10.1037/ser0000146. 29120210 10.1037/ser0000146

[26] Mendoza H, Masuda A, Swartout KM. Mental health stigma and self-concealment as predictors of help-seeking attitudes among Latina/o college students in the United States. Int J Adv Couns. 2015;37(3):207–22. 10.1007/s10447-015-9237-4.

[27] Pro G, Brown C, Rojo M, Patel J, Flax C, Haynes T. Downward national trends in mental health treatment offered in Spanish: State differences by proportion of Hispanic residents. Psychiatr Serv. 2022;73(11):1232–8. 10.1176/appi.ps.202100614. 35502519 10.1176/appi.ps.202100614

[28] Broman CL. Race differences in the receipt of mental health services among young adults. Psychol Serv. 2012;9(1):38–48. 10.1037/a0027089. 22449086 10.1037/a0027089

[29] Kawaii-Bogue B, Williams NJ, MacNear K. Mental health care access and treatment utilization in African American communities: an integrative care framework. Best Pract Ment Health. 2017;13(2):11–29.

[30] Hingwe S. Mental health considerations for Black, Indigenous, and people of color: trends, barriers, and recommendations for collegiate mental health. In: Riba MB, Menon M, eds. College Psychiatry: Strategies to Improve Access to Mental Health. Psychiatry Update. Springer International Publishing. 2021;85–96.

[31] Barksdale CL, Molock SD. Perceived norms and mental health help seeking among African American college students. J Behav Health Serv Res. 2009;36(3):285–99. 10.1007/s11414-008-9138-y. 18668368 10.1007/s11414-008-9138-y

[32] Center for Behavioral Health Statistics and Quality. 2016 National Survey on Drug Use and Health: Detailed Tables. Substance Abuse and Mental Health Services Administration; 2017. https://www.samhsa.gov/data/sites/default/files/NSDUH-DetTabs-2016/NSDUH-DetTabs-2016.pdf. Accessed 19 February 2024.

[33] Gone JP. The (post)colonial predicament in community mental health services for American Indians: explorations in alter-native psy-ence. Am Psychol. 2021;76(9):1514–25. 10.1037/amp0000906. 35266761 10.1037/amp0000906

[34] Goetz CJ, Mushquash CJ, Maranzan KA. An integrative review of barriers and facilitators associated with mental health help seeking among Indigenous populations. Psychiatr Serv. 2023;74(3):272–81. 10.1176/appi.ps.202100503. 36065579 10.1176/appi.ps.202100503

[35] Vora AS, Grilo SA. The influence of identity on multiracial emerging adults’ health and experiences seeking healthcare in the United States: a qualitative study. J Racial Ethn Health Disparities Published Online September 12, 2023. 10.1007/s40615-023-01785-1. Accessed 12 January 2024. 10.1007/s40615-023-01785-1 37698751

[36] Nakamura L, Jo D, Masuda A. Mental health help-seeking experience and attitudes in Asian American, multiracial American, and White American emerging adults. Int J Adv Couns. 2022;44(3):432–52. 10.1007/s10447-022-09470-4.

[37] McBain RK, Cantor J, Pera MF, Breslau J, Bravata DM, Whaley CM. Mental health service utilization rates among commercially insured adults in the US during the first year of the COVID–19 pandemic. JAMA Health Forum. 2023;4(1):e224936. 10.1001/jamahealthforum.2022.4936. 36607697 10.1001/jamahealthforum.2022.4936 PMC9857246

[38] Dunbar MS, Sontag-Padilla L, Kase CA, Seelam R, Stein BD. Unmet mental health treatment need and attitudes toward online mental health services among community college students. Psychiatr Serv Wash DC. 2018;69(5):597–600. 10.1176/appi.ps.201700402. 10.1176/appi.ps.201700402 29540117

[39] Grieco-Page H, Black CJ, Berent JM, Gautam B, Betancourt TS. Beyond the pandemic: leveraging rapid expansions in U.S. telemental health and digital platforms to address disparities and resolve the digital divide. Front Psychiatry. 2021;12. 10.3389/fpsyt.2021.671502. 10.3389/fpsyt.2021.671502 PMC8377496 34421669

[40] Kodish T, Lau AS, Gong-Guy E, et al. Enhancing racial/ethnic equity in college student mental health through innovative screening and treatment. Adm Policy Ment Health. 2022;49(2):267–82. 10.1007/s10488-021-01163-1. 34505211 10.1007/s10488-021-01163-1 PMC8850289

[41] Egan RP, Hurley DB, Goetz MC, Smith CS, Palmer BA, St. Hill CA. Disparities in mental health access before and after transitioning to telehealth. J Rural Ment Health. 2022;46(4):271–6. 10.1037/rmh0000214.

[42] Eberly LA, Khatana SAM, Nathan AS, et al. Telemedicine outpatient cardiovascular care during the COVID–19 pandemic. Circulation. 2020;142(5):510–2. 10.1161/CIRCULATIONAHA.120.048185. 32510987 10.1161/CIRCULATIONAHA.120.048185 PMC9126131

[43] R Core Team. R: a language and environment for statistical computing. Published online 2023. https://www.R-project.org/. Accessed 12 January 2024.

[44] Champely S. Pwr: Basic functions for R Power Analysis. Published online 2020. https://CRAN.R-project.org/package=pwr. Accessed 19 January 2024.

[45] McHugh ML. The chi-square test of independence. Biochem Med. 2013;23(2):143–9. 10.11613/BM.2013.018. 10.11613/BM.2013.018 PMC3900058 23894860

[46] Franke TM, Ho T, Christie CA. The chi-square test: often used and more often misinterpreted. Am J Eval. 2012;33(3):448–58. 10.1177/1098214011426594.

[47] Delucchi KL. On the use and misuse of chi square. In: A Handbook for Data Analysis in the Behavioral Sciences. Lawrence Erlbaum. 1993; pp. 294–319.

[48] Sharpe D. Chi-square test is statistically significant: now what? Pract Assess Res Eval. 2015;20:8. 10.7275/tbfa-x148.

[49] Agresti A. An Introduction to Categorical Data Analysis. 2nd ed. Wiley & Sons; 2007.

[50] Center for Collegiate Mental Health. 2023 Annual Report. Pennsylvania State University. https://ccmh.psu.edu/assets/docs/2023_Annual%20Report.pdf. Accessed 2 February 2024.

[51] Dallo FJ, Kindratt TB, Seaton R, Ruterbusch JJ. The disproportionate burden of COVID-19 cases among Arab Americans. J Racial Ethn Health Disparities. 2023;10(3):1108–14. 10.1007/s40615-022-01298-3. 35394622 10.1007/s40615-022-01298-3 PMC8992413

[52] Management and Budget Office. Revisions to OMB’s statistical policy directive no. 15: standards for maintaining, collecting, and presenting federal data on race and ethnicity. Office of Information and Regulatory Affairs, Office of Management and Budget, Executive Office of the President, 2024. https://www.federalregister.gov/documents/2024/03/29/2024-06469/revisions-to-ombs-statistical-policy-directive-no-15-standards-for-maintaining-collecting-and. Accessed 03 May 2024.

[53] Cabral RR, Smith TB. Racial/ethnic matching of clients and therapists in mental health services: a meta-analytic review of preferences, perceptions, and outcomes. J Couns Psychol. 2011;58(4):537–54. 10.1037/a0025266. 21875181 10.1037/a0025266

[54] Duncan LE, Johnson D. Black undergraduate students’ attitude toward counseling and counselor preference. Coll Stud J. 2007;41(3):696–720.

